# Discussions of the Michigan State Dental Association

**Published:** 1860-03

**Authors:** 


### Proceedings of Societies.

### DISCUSSIONS OF THE MICHIGAN STATE DENTAL ASSOCIATION.

The above named association met at Firemen's Hall, in the city of Detroit, on Tuesday evening, January 10th, 1860.

After reading the minutes of last meeting, election of officers for the ensuing year and election of new members, the President elect, Dr. C. S. Chittenden, of Hamilton, C. W., delivered a short and appropriate address, thanking the Association for the honor conferred, encouraging them to still greater efforts in the good work of diffusing useful knowledge, and, by frequent social intercourse, to break down the professional prejudices, and thereby unite and harmonize for the elevation of the profession.

Dr. White read an able essay on professional intercourse and practice, which was requested for publication, but we have not been supplied with a copy.

Dr. Atkinson, of Cleveland, Ohio, made some very appropriate remarks, drawing parallels between the proper spherer and relative positions of the Medical and Dental professions; stating that the Medical profession have always stood aloof and placed a barrier between themselves and the Dentist, but at last the Dental profession have succeeded in pecking a hole through the wall so that the divine light may pass through; at last the curtain is removed and the divine light permitted to shine in upon the soul.

It has been common to call the Dentist a "Tooth Doctor;" there is no more propriety in this, than being called a doctor of any other specialty. If we take the tooth we also take the soul of it. Knowledge is *divine*; it is not yours, nor mine, nor does it belong to any one in particular but to God, —it is his—and he diffuses it to all who will honestly and in truth receive it. We have no right to claim any thing as our own; but when a new thought strikes us, let us say: "Freely have I received and freely will I give."

Physicians presume that dentists have stolen from them their profession, like an old maid, whose affections have gone out and finding nothing to cling to have returned into the heart and dried up. Physicians have themselves stolen every thing. The old Africans, if alive, could tell us things we never dreamed of. Let us be honest examples, nor claim that which is not ours, but freely give credit to those who have more knowledge than ourselves. Every thing in the earthy sphere are aggregates of humanity and have an elective affinity; every thing has in it imprisoned something to enrich us. If we should eat an entire ox we would probably feed the ox instead of being fed, but we cut it in pieces and are nourished thereby,—we can not aggregate to ourselves life principles without death to something else.

Take an apple, bite it, and at the moment it rubs upon the teeth and the saliva flows upon it, the life of the apple is destroyed and we are nourished.

We should be examples to our patients as we are to our children, who asking for a penny to get candy, we give it and direct them to get such as has no color; we do so to gratify the child and thus gratify ourselves, but find he is not satisfied. You refuse to give more, having a higher appreciation of the child's good than it has of itself. We should be examples in cleanliness, in eating, and in the care of our teeth. No *dirty* man can be in affinity with the *divine.* According to Scripture each of us is of more value than many sparrows. If we could have hold of every man and woman who are about to have a child, we would produce *perfect* specimens of humanity, and in two generations would have all in harmony.

To be *men* we must be well *begotten*, well *gestated* and well *born.* There are positive and negative forces in life; if we are pure males we are purely positive; if we are pure females we are purely negative. Every science has its center from which all light emanates; our profession is the center of all sciences. If we were perfect we would have wings and soar through space just as our thoughts do; this is stong language but true. Paul was a good man but had to be knocked down before he could comprehend the truth.

A paper from Dr. Bancroft on Difficult Dentition was read and ordered to be published. (See Original Communications.)

### First Subject.—*Difficult Dentition.*

Dr. Cahoon thought the subject too broad. A child is brought to the dentist, teeth not developed in time on account of deficiency of phosphate of lime; if we give good food, good air and keep clean, we will have good teeth.

Dr. Benedict is willing to agree that if we all lived first as we should, there would be no difficulty. No need of lawyers, physicians or dentists, but such is not the case, and these do exist and are useful in assisting each other.

We must take things as we find them. Nature will *generally* do the work of dentition, but not always; she sometimes needs assistance.

Oft times the mother presents herself to the dentist with a child, whose gums are swollen and inflamed. What shall we do in such cases? Some would use the lancet, others not; has children himself, and one thing is always done. Where the gums show symptoms of inflammation and swelling, rubs gently with the finger several times a day, in consequence of which the inflammation subsides after rubbing a few times; in removing the finger the child will itself grasp and replace it in the mouth. The presumption is that the teeth are pressed into the gum and the inflammation is dissipated. Would not lance the gums in any case except one of great suffering, or where the child is likely to have spasms. Mild physic will sometimes relieve, but in severe cases would cut through the gums to the teeth, which would relieve the pressure and consequent inflammation.

Dr. Porter does not consider that it is the amount of blood removed that relieves, but the cutting gives relief from pressure; so marked is this that children will sometimes open the mouth and desire the cutting.

Dr. Atkinson: The proper ratio between loss and supply of the tissues is not kept up. The absorbing power may be restored, and the tooth irrupted without difficulty.

Never cut with the expectation of irrupting the teeth until you see the white, transparent appearance of the tooth, or a dark congested appearance. In such cases always cut, and would rather cut ten times when not needed, than to fail to cut once when it is required. It is a false theory that a cicatrix is more difficult to absolve than healthy tissue.

Dr. White: The management of children during dentition is very important; it is painful to see how foolishly and injudiciously children are generally treated; it will tell through life on the destiny of the child. There is much fault in regard to food during dentition.

Dr. Farmer: Youngest member of the profession by odds, don't see the propriety of going back so far; we can't go about and instruct people when about to commit a certain act, to bear certain things in mind, that they may produce children with good teeth. Likes the remarks of Dr. Atkinson. When a white spot is observed on the coming tooth, or a dark purple tinge, would not hesitate to cut; but prefers to do so with a finger or thumb nail, as the bleeding is more easily stopped.

It is conceded that impressions can be made on a child in utero as early as the fourth month; if it can be done at the fourth month it can be done earlier; but we have to do with cases as they are presented, and not with fancies.

Dr. CHITTENDEN has had a little difficulty in one family. We have but little to do with the matter when the mother is healthy. The dentist is not called upon till there is some difficulty. Thinks the difficulty occurs from an insufficiency of calcareous elements in the tooth. Is a Homœopathist and employs no other physician. When a child is suffering intensely, and is in danger of going into convulsions, recommends the use of Cal. Carb. from the use of which relief is rapidly obtained; in similar cases use the same remedy; children will readily take it, it being pleasant. When white, or purple spots appear on the tooth, would cut.

SECOND SUBJECT.—*Effects of Diseased Teeth and Gums on the General Health.*

Dr. ATKINSON: Two cases, Mrs. S. of Newark, Ohio, for years under necessity, as she thought, of wearing a seaton in her side, for supposed consumption. While she could keep up discharge from seaton, improved. Seaton dried up, increased discharge from lungs; meantime she met another lady, also wearing a seaton. One of them had the toothache, both have lived in same way by advice of physician Met Mrs. S. and told her if she would have her mouth cleansed out there would be no necessity for seatons; after some delay she had her teeth extracted, and mouth cleansed and a set of artificial teeth. Seaton dried up, and discharge from lungs ceased, and following up the case for several years, the patient became entirely well.

Mrs. I. similar, only several molar teeth in the mouth. Lady stooped and could not walk without assistance. She became as well and strong as her neighbor, not suspected of having consumption.

Dr. CAHOON: Remembers a similar but not so aggravated a case, under care of physician. Was called in to examine, found several roots of teeth quite covered up, and diseased antrum, removed roots, and with but little medication got well.

Dr. PORTER. Neighbor very unwell, going about with over-coat all summer, unable to do any thing and supposed to be in consumption. Came and requested a tooth extracted. Removed several and patient was soon restored to perfect health. Another similar, with throat very much inflamed.

Dr. MANSFIELD. Lady patient suffered severely for several months, face swollen and tied up all the time. Suffered much pain, teeth decayed and considerable absorbtion; in extracting two came out together, bringing with them a large piece of the process.

Dr. ATKINSON wished to be correct in terms; it could not be the process unless secondary growth. Has a case in which three teeth joined together by an osseous union; the periosteum is the bone making organ. Was requested to describe the condition of anchyllosis and necrosis, which he did in his usual clear, minute, vigorous and scientific manner.

Dr. CHITTENDEN has had several cases. Rev. Dr. G.'s wife supposed to be in consumption, catamenial discharges ceased; all teeth loose except two or three; the lady insisted upon having all the teeth out. Doctor was much affected in contemplation of the loss of his wife. Dr. C. thought it not worth while to put in an artificial denture, but the lady insisted, it was done, and in less than six months was as healthy as any body.

Dr. FARMER: Lady had teeth filled and in about six weeks after returned in great distress, desiring to have the teeth extracted; told her she must go elsewhere, teeth all right, and would not take them out. After some inquiry discovered her condition; prescribed some simple remedies, which brought on catamenial discharge, and sent her home next day. She came in as bright as a dollar. The difficulty was simply suppression of menses.

Another case of same kind; was sent for in great haste. Great suffering and swelling, applied leeches, and a dose of citrate of magnesia, and administered the following prescription, which he considers of great value in such cases:

℞ Syrup Rhei—℥ ij

Sulph. Quinine—℈ ij

Fowler's Solution—Gtts. xlv.

Dose—1 teaspoonful three times a day.

Extracted tooth at the first call and urgent solicitation of patient; never will do so again.

Dr. CAHOON also described two or three very interesting cases so nearly like the above, that a special description is not necessary.

Dr. BENEDICT also reported an aggravated case in which nine teeth were removed; within four weeks after the operation the general health was entirely restored. Another case in which the lady was sick and in bed: examined the teeth, found the mouth full of old roots; recommended extraction and medical treatment. Next day removed the roots, and the day following cleansed the teeth, since which time the health has rapidly improved.

THIRD SUBJECT.—*The Proper Treatment of Diseased Gums.*

Dr. WHITING finds diseased gums to proceed generally from diseased roots and tartar. Sometimes from scrofulous and syphilitic diathesis. When from the former cause, remove the roots and tartar and the mouth will get well itself, when from the latter cause, constitutional treatment is required.

Dr. Cahoon finds uncleanliness the general cause of disease; uncleanliness generates tartar, which is insinuated under the free margin of the gums, causes absorption. Sometimes the acids and fluids of the mouth will so irritate and inflame as to keep the gums sore and diseased all the time. Tincture of Rhatany is probably the best remedy as a lotion, after the removal of the tartar or diseased roots. Though one may tell another what should be done, every one must see the case and judge for himself; if the cause is constitutional, then constitutional treatment must be resorted to; Tincture of arnica is also a good and efficient local remedy.

Dr. Porter agrees with Dr. Cahoon, ascertain and remove the cause and the case is easily cured; uses a great variety of remedies; tincture of rhatany, arnica, creosote, chlorid of zinc, etc.

Dr. Farmer wishes to know what should be done where the festoons of the gums shoot up like so many parasites, in a high state of inflammation.

Dr. Mansfield says cut them off.

Dr. Cahoon applies nitrate of silver; they *must* be removed; if this does not bring them down then cuts them off.

Dr. Atkinson, at considerable length, entered into the pathological and physiological condition generally; deem such, as fungus growth, and with a sharp knife lops them off; if in haste, use styptics, after that creosote and chlorid of zinc; creosote first, if cut off from all but two remedies, would select creosote and chlorid of zinc, *give me these.*

Dr. Benedict followed in a few very able remarks, on the same subject, with dissertation on the use of the tooth-brush, advancing some valuable hints, which he has promised to write out, and furnish for publication in the *Register.*

FOURTH SUBJECT.—*Mechanical Dentistry.*

Dr. PERINE describes the process of Dr. C. Sill, of Pittsburg, for obtaining a correct metallic cast, by taking the impression in plaster. He places upon a stone to draw off moisture, while the metal is melting. Surround the impression with a metal ring, stir the metal well and pour carefully into the impression. Babbit metal is best. Dr. P. also recommends a solution of silicate of soda, for repairing plaster casts, and for filling up between the points of teeth, in rubber work, to prevent the rubber from forcing and showing between the teeth. Gutta Percha was also suggested by Dr. Atkinson, and commented upon by several members; in this it is necessary for the plaster to be poured immediately on the removal of the impression from the mouth.

Dr. PORTER exhibited a cast of a case of artificial palate; plate was fitted, and speech restored to great extent. This was a very interesting and difficult case.

Dr. ESTILE, of Toledo, Ohio, sent in some specimens of metal for plates and substitute for silver, being of much finer texture and resisting the action of acids and secretions of the mouth. On one of the strips pure copper was melted, and on the other a piece of a Spanish twenty-five cent piece, without change of color in the metals.

Dr. E. also stated that he had it in the mouth for several months, wearing bright and clear. Dr. E. proposes to manufacture it and supply the profession at about two dollars and a half per ounce.

MISCELLANEOUS.

Dr. ATKINSON, on the use of the mallet, says: If you want to save teeth, save pain and save time, use the mallet; and the time is not far distant when every dentist will use it. He then gave a detailed account of how he came to use it, his success and experience.

